# Determinants of skilled attendance for delivery in Northwest Ethiopia: a community based nested case control study

**DOI:** 10.1186/1471-2458-13-130

**Published:** 2013-02-12

**Authors:** Zelalem Birhanu Mengesha, Gashaw Andargie Biks, Tadesse Awoke Ayele, Gizachew Assefa Tessema, Digsu Negesse Koye

**Affiliations:** 1 Department of Reproductive Health, Institute of Public Health, University of Gondar, Gondar, Ethiopia; 2 Department of Health Service Management and Health Economics, Institute of Public Health, University of Gondar, Gondar, Ethiopia; 3 Department of Epidemiology and Biostatistics, Institute of Public Health, University of Gondar, Gondar, Ethiopia

**Keywords:** Determinant, Skilled birth attendance, Ethiopia

## Abstract

**Background:**

The fifth Millennium Development Goal calls for a reduction of maternal mortality ratio by 75% between 1990 and 2015. A key indicator to measure this goal is the proportion of births attended by skilled health personnel. The maternal mortality ratio of Ethiopia is 676 deaths per 100,000 live births. Skilled birth attendance is correlated with lower maternal mortality rates globally and in Sub-Saharan Africa. However, the proportion of births with a skilled attendant is only 10% in Ethiopia. Therefore identifying the determinants of skilled attendance for delivery is a priority area to give policy recommendations.

**Methods:**

A community based nested case control study was conducted from October 2009 – August 2011 at the University of Gondar health and demographic surveillance systems site located at Dabat district, Northwest Ethiopia. Data were obtained from the infant mortality prospective follow up study conducted to identify the determinants of infant survival. A pretested and structured questionnaire via interview was used to collect data on the different variables. Logistic regression analysis was used to identify the determinants of skilled birth attendance. Strength of the association was assessed using odds ratio with 95% CI.

**Results:**

A total of 1065 mothers (213 cases and 852 controls) were included in the analysis. Among the cases, 166 (77.9%) were from urban areas. More than half (54%) of the cases have secondary and above level of education. Secondary and above level of education [AOR (95%CI) = 2.8 (1.29, 3.68)] and urban residence [AOR (95%CI) = 8.8 (5.32, 14.46)] were associated with skilled attendance for delivery. Similarly, women who had ANC during their pregnancy four or more times [AOR (95%CI) = 2.8 (1.56, 4.98)] and who own TV [AOR (95%CI) = 2.5 (1.32, 4.76)] were more likely to deliver with the assistance of a skilled attendant.

**Conclusions:**

Women’s education, place of residence, frequency of antenatal care visit and ever use of family planning were found to be determinants of skilled birth attendance. Encouraging women to complete at least secondary education and to have antenatal care frequently are important to increase skilled attendance during delivery.

## Background

One of the eight Millennium Development Goals (MDGs) adopted by United Nations in September 2000 was improving maternal health. Despite proven interventions that could prevent disability or death during pregnancy and childbirth, maternal mortality remains a major burden in many developing countries. The Maternal Mortality Ratio (MMR), the most common measure of maternal health, continues to be a major challenge in Africa compared to performance in the rest of the world [1,2].

In 2008, of the estimated 358 000 maternal deaths worldwide, developing countries accounted for 99% (355 000). Nearly three fifths of the maternal deaths (204 000) occurred in the Sub- Saharan Africa (SSA) region alone, followed by South Asia (109 000). Together the two regions accounted for 87% of such deaths globally. Southern Asia has made steady progress, with a 53% decline in maternal mortality between 1990 and 2008. In contrast, the ratio has fallen by only 26% in Sub-Saharan Africa, though evidence suggests that progress has picked up speed since 2000. More than 50% of all maternal deaths were in only six countries in 2008 (India, Nigeria, Pakistan, Afghanistan, Ethiopia, and the Democratic Republic of Congo) [1,3,4]. According to the 2011 Ethiopian Demographic and Health Survey (EDHS) report, MMR was 676 deaths per 100,000 live births. Maternal deaths represent 30% of all deaths to women age 15–49, compared with 21% in the 2005 EDHS and 25% in the 2000 EDHS [5-7].

The fifth MGD calls for a reduction in the maternal mortality ratio by 75% between 1990 and 2015. A key indicator to measure this goal is the proportion of births attended by skilled health personnel [8]. A skilled birth attendant is an accredited health professional – such as a midwife, doctor or nurse – who has been educated and trained to proficiency in the skills needed to manage normal (uncomplicated) pregnancies, childbirth and the immediate postnatal period, and in the identification, management and referral of complications in women and newborns [9]. A skilled health professional can administer interventions to prevent and manage life-threatening complications, such as heavy bleeding, or refer the patient to a higher level of care when needed [10].

The presence of a trained health-care worker during delivery is crucial in reducing maternal deaths whereby estimates between 13%–33% of maternal deaths could be averted by the presence of a skilled birth attendant [11]. Skilled birth attendance is also correlated with lower maternal mortality rates in SSA [12]. Proportion of deliveries attended by skilled health personnel in developing regions overall rose from 55% in 1990 to 65% in 2009. However, the proportion in Ethiopia is very much lower than SSA. Even for women who have access to the services, the proportion of births with a skilled attendant is very low. Skilled care at delivery is usually provided at health facilities (hospitals and health centers) in Ethiopia. One of the targets of the Ethiopian reproductive health strategy is to increase the proportion of births attended by skilled health personnel either at home or in a facility to 60% by 2015 [13]. However, the 2011 EDHS revealed that only 10% of births were delivered by the assistance of a skilled attendant [5].

Therefore identifying the determinants of skilled attendance for delivery is a priority area to give policy recommendations.

## Methods

A community based nested case control study was conducted from October 2009-August 2011 at the University of Gondar health and demographic surveillance systems site which is located at Dabat district, Northwest Ethiopia. Dabat district is located about 75 kms away from Gondar town. It has an area of 1,199.15 km^2^ and had an estimated total population of 145,458 (89% live in rural area). Altitude ranges from 1000–2600 m above sea level. Agriculture is the backbone of their economy. The district has a total of 29 functioning health institutions (27 health posts and 2 health centers).

The demographic survey site has three ecological regions including high, mid and lowland. Baseline data collection for the research center was started in November 1996 and subsequent vital events (birth, death, migration) registration has been continued since August 2004. According to the second baseline survey conducted in August 2007, the total numbers of households were about 9,334 and the total population of the center was 46,165. Antenatal care coverage and prevalence of contraceptive were found to be 51% and 19.2% respectively. Only 7.2% of deliveries took place in health institutions and 7.85% of the births were attended by skilled professionals [14].

All mothers with second and third trimester pregnancy were registered and on follow up from October 2009 to August 2011 for infant mortality prospective study. The population for this study consisted of sampled cases and controls of women who gave birth during the follow up period. Cases were women who gave birth by the help of skilled attendants while controls were those who gave birth by the help of traditional birth attendants, relatives or health extension workers. All cases and a random sample of controls were taken from the deliveries, which took place in the study period.

Data were collected via interview using pretested and structured questionnaire. The questionnaire was prepared in English, translated to Amharic and then translated back to English to check for consistency. Six female data collectors working at the research site were recruited. Training was given to the data collectors for one day about the objective, relevance of the study, confidentiality of information, participant’s right, pre-test, informed consent and techniques of interview.

The sample size was calculated using Epi info version 3.3.2.0 by considering the following assumptions: the proportion of urban women among the controls 23.9% [15], 95%CI, 80% power and case to control ratio of 1:4 to detect an odds ratio of 2.0. The total sample size was 510(102 cases and 408 controls). There were 1752 pregnant women recruited for the prospective infant mortality study. Among these, 213 (12.2%) delivered with the assistance of a skilled attendant. Since there were more participants for the study from the follow up database, we took all the cases (213) and a random sample of controls (852 out of the 1539) for final analysis.

Before the actual data collection, pretest was conducted in adjacent kebeles to ensure the validity of the survey tool. The supervisors and the principal investigator made frequent checks on the data collection process to ensure the completeness and consistency of the gathered information.

Data were entered and cleaned using EPI info version 6 statistical software and exported to SPSS version 16 statistical packages for analysis. Frequencies, proportion and summary statistics were used to describe the study population in relation to relevant variables.

### Hypothesis

Factors affecting utilization of skilled attendants are the same in Dabat and elsewhere in Ethiopia. These are parity, having ANC, women`s and their husbands educational status, occupational status, having TV and Radio, urban residence, history of family planning use, younger age, economic status, knowledge about danger signs of pregnancy, availability and attitude towards health professionals, husband`s approval and involvement.

Bivariate and multivariate analyses were carried out to see the effect of each independent variable on the dependent variable. Odds ratio with 95%CI was computed to assess the strength of the association and statistical significance. P-value less than 0.05 was used to declare statistical significance in the multivariate analysis.

Ethical clearance was obtained from the Institution Review Board of the University of Gondar. The purpose and importance of the study were explained to the participants. Data were collected after full informed verbal consent is obtained and confidentiality of the information has been maintained throughout by excluding names as identification in the questionnaire and keeping their privacy during the interview by interviewing them alone.

## Results

### Socio demographic characteristics of the participants

A total of 1065 mothers (213 cases and 852 controls) were included in the analysis. Among the cases, 166(77.9%) were from urban areas. More than half (54%) of the cases have secondary and above level of education. Around 89 and 98% of the controls did not have radio and television respectively. Orthodox religion followers accounted 94.4% of the case and 98.9% of the controls. Married participants accounted 83.6% and 94.6% among the cases and controls respectively. One hundred eleven (52.1%) of the cases and 770 (90.4%) of the controls were housewives by occupation. Secondary and above level of education was attained by 94 (44.1%) among the cases’ husbands. Ninety nine (46.5%) and 764 (89.7%) of the cases’ and controls’ husbands were farmers [Table 1].

**Table 1:** Socio demographic characteristics of the study participants, Dabat DSS site, Northwest Ethiopia, 2011 (n = 1065)

**Variables**	**Category**	**Skilled birth attendance**	
		**Cases (%)**	**Controls (%)**
Residence	Urban	166 (77.9)	87 (10.2)
	Rural	47 (22.1)	765 (89.8)
Own Radio	Yes	113 (53.1)	97 (11.4)
	No	100 (46.9)	755 (88.6)
Own TV	Yes	66 (31.0)	18 (2.1)
	No	147 (69.0)	834 (97.9)
Maternal education	Can not read and write	69 (32.4)	673 (79.0)
	Primary	29 (13.6)	106 (12.4)
	Secondary and above	115 (54.0)	73 (8.6)
Religion	Orthodox	201 (94.4)	843 (98.9)
	Muslim	12 (5.6)	9 (1.1)
Marital status	Married	178 (83.6)	806 (94.6)
	Single/widowed/separated	35 (16.4)	46 (5.4)
Maternal occupation	Farmer	2 (.9)	13 (1.5)
	Own business	51 (23.9)	20 (2.3)
	House wife	111 (52.1)	770 (90.4)
	Others	49 (23.0)	49 (5.8)
Maternal age	≤19	18 (8.5)	102 (12.0)
	20-34	155 (72.8)	597 (70.1)
	≥35	40 (18.8)	153 (18.0)
Husband education	Can not read and write	65 (30.5)	534 (62.7)
	Primary education	54 (25.4)	246 (28.9)
	Secondary and above	94 (44.1)	72 (8.5)
Husband occupation	Student	4 (1.9)	29 (3.4)
	Farmer	99 (46.5)	764 (89.7)
	own business	50 (23.5)	32 (3.8)
	Others	60 (28.2)	27 (3.2)

### Obstetric characteristics of the study participants

One hundred fifty two (71%) of the cases had ANC follow up but 81.3% of the controls didn’t have. Nearly two third (61.5%) of the cases ever used family planning methods and only 19.8% of the controls ever use family planning. Both the cases and controls had almost similar history of abortion and still birth. Three quarters (76.8%) of the controls delivered at home but 90.6% of the cases had delivered at health institutions. Nearly one third of the cases (37.1%) had no history of prior pregnancy but only 15.4% of the controls had more than one pregnancy history [Table 2].

**Table 2:** Obstetric histories of the participants at Dabat DSS site, Northwest Ethiopia, 2011 (n = 1065)

**Variable**	**Category**	**Skilled birth attendance**	
		**Cases**	**Controls**
ANC follow up	Yes	152 (71.4)	159 (18.7)
	No	61 (28.6)	693 (81.3)
Ever use of family planning	Yes	131 (61.5)	169 (19.8)
	No	82 (38.5)	683 (80.2)
History of abortion	Yes	11 (5.2)	39 (4.6)
	No	202 (94.8)	813 (95.4)
History of still births	Yes	9 (4.2)	25 (2.9)
	No	204 (95.8)	827 (97.1)
Place of delivery	Home	16 (7.5)	654 (76.8)
	Hospital	42 (19.7)	0 (0)
	Health center/clinic	151 (70.9)	3 (0.4)
	Other government health institutions	4 (1.9)	195 (22.9)
Parity	1	79 (37.1)	131 (15.4)
	2-4	88 (41.3)	376 (44.1)
	≥5	46 (21.6)	345 (40.5)

### Determinants of skilled birth attendance

As clearly depicted on the multivariate logistic regression, urban residence, having TV, having ANC follow up, ever use of family planning and secondary and above level of maternal education were significantly and independently associated with skilled birth attendance.

Accordingly, women from urban areas were 8.8 times [AOR (95%CI) = 8.8 (5.32, 14.46)] more likely to have skilled birth attendance as compared to rural women. Those women who own TV were 2.5 times [AOR (95%CI) = 2.5 (1.32, 4.76)] more likely to utilize skilled birth attendance as compared to their counter parts.

Having ANC follow up was found to be an important determinant of skilled birth attendance in which women who had four or more visits were 2.8 times [AOR (95%CI) = 2.8 (1.56, 4.98)] more likely to have skilled birth attendance as compared to those who didn’t have follow up.

Regarding family planning use, ever users were 1.71 times [AOR (95%CI) =1.71(1.09, 2.69)] more likely to have skilled birth attendance as compared to those who didn’t. Women with secondary and above education were about 2.18 times [AOR (95%CI) =2.18 (1.29, 3.68)] more likely to have skilled attendance than those with no formal education. However, having radio, husband’s educational status, marital status, maternal age and husband’s occupation were not significantly associated with skilled birth attendance [Table 3].

**Table 3:** Unadjusted and adjusted odds ratios (OR) and 95% confidence intervals (CI) of determinants of skilled birth attendance at Dabat DSS site, Northwest Ethiopia, 2011 (n = 1065)

**Determinants**		**Cases**	**Controls**	**Crude OR (95%CI)**	**Adjusted OR (95%CI)**
**Residence**	Urban	166 (77.9)	87 (10.2)	31.1 (20.96, 45.98)	8.8 (5.32, 14.46)
	Rural	47 (22.1)	765 (89.8)	1	1
**Own radio**	Yes	113 (53.1)	97 (11.4)	8.8 (6.25, 12.38)	
	No	100 (46.9)	755 (88.6)	1	
**Own TV**	Yes	66 (31.0)	18 (2.1)	20.8 (12.004, 36.052)	2.5 (1.32, 4.76)
	No	147 (69.0)	834 (97.9)	1	1
**ANC follow up**	No	152 (71.4)	159 (18.7)	1	1
	1-3	61 (28.6)	693 (81.3)	5.9 (3.92, 8.82)	1.6 (0.94, 2.73)
	≥4	152 (71.4)	159 (18.7)	20.2 (13.06, 31.07)	2.8 (1.56, 4.98)
**Family planning use**	Yes	131 (61.5)	169 (19.8)	6.5 (4.67, 8.92)	1.71 (1.09, 2.70)
	No	82 (38.5)	683 (80.2)	1	1
**Husband`s education**	Illiterate	65 (30.5)	534 (62.7)	1	
	Primary	54 (25.4)	246 (28.9)	1.8 (1.22, 2.67)	
	Secondary and above	94 (44.1)	72 (8.5)	10.7 (7.19, 16.01)	
**Maternal education**	Illiterate	69 (32.4)	673 (79.0)	1	1
	Primary	29 (13.6)	106 (12.4)	2.7 (1.65, 4.31)	1.23 (0.67, 2.27)
	Secondary and above	115 (54.0)	73 (8.6)	15.4 (10.47, 22.56)	2.18 (1.29, 3.68)
**Marital status**	Married	178 (83.6)	806 (94.6)	0.3 (0.18, 0.46)	
	Single/Widowed/Separated	35 (16.4)	46 (5.4)	1	
**Maternal age**	≤19	18 (8.5)	102 (12.0)	0.7 (0.37, 1.24)	
	20-34	155 (72.8)	597 (70.1)	0.9 (0.67, 1.47)	
	≥34	40 (18.8)	153 (18.0)	1	
**Husbands occupation**	Student	4 (1.9)	29 (3.4)	1	
	Farmer	99 (46.5)	764 (89.7)	0.1 (0.02, 0.19)	
	Own business	50 (23.5)	32 (3.8)	0.1 (0.04, 0.10)	
	Others	60 (28.2)	27 (3.2)	0.7 (0.37, 1.33)	
**Parity**	1	79 (37.1)	131 (15.4)	4.5 (2.99, 6.85)	
	2-4	88 (41.3)	376 (44.1)	1.8 (1.19- 2.58)	
	≥5	46 (21.6)	345 (40.5)	1	
**Religion**	Orthodox	201 (94.4)	843 (98.9)	0.2 (0.07, 0.43)	
	Muslim	12 (5.6)	9 (1.1)	1	

## Discussion

Majority of deaths from obstetric complications are preventable and that every pregnancy faces risk which may not always be detected through the risk assessment approach during ANC [15]. Delivery assisted by skilled providers is the most important proven intervention in reducing maternal mortality and one of the MDG indicators to track national effort towards safe motherhood [16]. However, the proportion of births attended by skilled health personnel did not increase substantially from 2000 to 2011 in Ethiopia i.e. it increased from 5% to 10%. This is also reflected by the high maternal mortality ratio observed in the 2011 EDHS [5]. In this study, a number of socio demographic and other factors were found to have a significant influence on the use of skilled care at delivery. These include residence, maternal education, owning TV and number of ANC visits.

Antenatal care with a skilled provider is one of the interventions that reduce maternal mortality. Because it allows early detection of obstetric complications and gives an opportunity to influence women`s decision to have a skilled attendance during child birth. WHO recommends at least four visits starting from the first trimester. Interestingly, antenatal care was a significant positive determinant of skilled attendance during delivery only for those women who attended four or more times. This finding is consistent with other studies done in Tanzania and Cambodia [17,18].

This study showed the importance of women’s education for the utilization of skilled birth attendance. Women with secondary and above education were about two times more likely to have skilled attendance than those with no formal education. This finding is in line with most maternal and child health studies conducted in developing countries [15,19,20]. The effect of education on the use of skilled birth attendance can be explained in various ways. Educated women have higher level of health awareness and greater knowledge of available health services. They have also improved ability to afford the cost of health care, and their enhanced level of autonomy results in improved ability and freedom to make health-related decisions, including choice of maternal services to use [21,22]. It is also likely that educated women will seek higher quality services and have greater ability to use health care inputs that offer better care [22]. Better educated women are more likely to have knowledge of the long-term benefits of the utilization of skilled maternity care services for both mother and babies compared to less educated women [23].

At the community level, urban residence was found to be associated with increased utilization of skilled birth attendants. This finding is in agreement with previous studies in sub Saharan Africa [15,24] and elsewhere [25,26] which have reported a significantly higher use of services in urban compared to rural areas. The reason might be urban women tend to have better access to health facilities and other promotional activities that are usually urban based. Moreover, rural women are more readily influenced by traditional practices that run contrary to modern health care [27].

Studies have shown that exposure to mass media promotes health-related behaviors including reproductive health services [28,29]. In line with this, women who own TV were more likely to utilize skilled birth attendance as compared to those who do not have. A study done in Bolivia also suggested that mass media are effective in information dissemination which fosters inter-personnel communication that could facilitate behavioral changes allowing for the adoption of new/different behaviors [30].

As a limitation, unmeasured independent factors may have important impacts on the outcomes that could not be captured in this study. Specifically, economical status, knowledge of danger signs, attitude towards health professionals and husbands` approval and involvement. This is because of the nature of the study that a secondary analysis of a prospective infant mortality study is done to identify the determinants of skilled birth attendance utilization.

## Conclusions

The results of this study showed that the determinants of skilled attendance during child birth are mostly socio-demographic and service related. The major factors identified include women’s education, place of residence, frequency of antenatal care service, ever use of family planning and having Television. Encouraging women to complete at least secondary school and teaching/encouraging women to have antenatal care frequently are important to increase skilled attendance during delivery. Researchers should also explore effective ways of increasing service utilization among less educated and uneducated women, women in rural areas and women who are not taking ANC.

## Competing interests

The authors declare that they have no competing interests.

## Authors’ contributions

ZB, GA, DN, TA, and GA participated in all steps of the study from its commencement to write up. They have reviewed and approved the submission of the manuscript.

## Pre-publication history

The pre-publication history for this paper can be accessed here:

http://www.biomedcentral.com/1471-2458/13/130/prepub
